# Cutaneous T‐cell lymphomas and dupilumab for atopic dermatitis: A systematic review and expert consensus

**DOI:** 10.1111/jdv.70407

**Published:** 2026-03-12

**Authors:** Florent Amatore, Madeleine Neildez, Adèle de Masson, Maxime Battistella, Marie Tauber, Saskia Ingen‐Housz‐Oro, Catherine Droitcourt, Delphine Staumont‐Sallé, Marie Beylot‐Barry

**Affiliations:** ^1^ Department of Dermatology, North Hospital, Assistance‐Publique Hôpitaux de Marseille, INSERM U1068 Aix‐Marseille University Marseille France; ^2^ French Cutaneous Lymphoma Group (GFELC) From Société Française de Dermatologie (SFD) Paris France; ^3^ Department of Dermatology, CHU Rennes, Univ Rennes, Inserm, EHESP Irset (Institut de Recherche en santé, Environnement et Travail) UMR_S Rennes France; ^4^ Department of Dermatology, Saint‐Louis University Hospital, AP‐HP, Université Paris Cité, INSERM U1342 Institut de Recherche Saint‐Louis Paris France; ^5^ Pathology Department Hôpital Saint‐Louis, AP‐HP Paris France; ^6^ Université Paris Cité, INSERM U1342, Institut de Recherche Saint‐Louis Paris France; ^7^ Groupe de Recherche sur L'eczéma ATopique (GREAT), from Société Française de Dermatologie (SFD)‐FRench Atopic DErmatitis Network (FRADEN) Network Paris France; ^8^ Department of Allergology and Clinical Immunology, Hospices Civils de Lyon, INSERM U1111, Centre International de Recherche en Infectiologie (CIRI) Lyon 1 University Lyon France; ^9^ Department of Dermatology Henri‐Mondor University Hospital, AP‐HP, Univ Paris Est Créteil, EpiDermE Créteil France; ^10^ Department of Dermatology, CHU de Lille, INSERM U1286 Inflammation Translational Research Institute (INFINITE), Univ Lille Lille France; ^11^ Department of Dermatology, CHU de Bordeaux, BoRdeaux Institute of onCology (BRIC), INSERM U1312 University of Bordeaux Bordeaux France

**Keywords:** atopic dermatitis, cutaneous T‐cell lymphomas, Dupilumab, interleukin 13, mycosis fungoides, Sézary syndrome, systematic review

## Abstract

**Introduction:**

Dupilumab, a standard treatment for atopic dermatitis (AD), has been associated with cutaneous T‐cell lymphomas (CTCL), particularly mycosis fungoides (MF) and Sézary syndrome (SS). Nevertheless, the available data remain heterogeneous.

**Objectives:**

To characterize the clinical features, timeline and outcomes of dupilumab‐related CTCL emergence in order to develop consensus‐based recommendations for dupilumab use in CTCL‐related settings.

**Methods:**

A systematic review was conducted involving 51 studies reporting cases of CTCL in patients treated with dupilumab, followed by a modified delphi process to generate expert consensus recommendations.

**Results:**

Data were obtained from 547 patients (mean age: 58 years; SD: 11.5; range: 10–85). New or worsening skin lesions were reported after dupilumab initiation, occurring at a mean of 8.9 months (SD 5.1; range 0.5–27 months). These were diagnosed as MF in 72% of cases, SS in 11% and other CTCL subtypes in 19%, of which 53% were at an early stage (stage ≤IIA). Dupilumab was discontinued in 75% of cases, and 62% received CTCL‐directed treatment. Clinical remission was achieved in 44 of the 63 patients with available follow‐up. The expert panel reached broad consensus, agreeing that dupilumab may unmask or exacerbate pre‐existing CTCL and should be avoided in cases of MF/SS and mogamulizumab‐induced rashes. They emphasized the importance of diagnostic vigilance, providing recommendations for skin biopsy, histology and clonality testing before and during treatment, particularly for patients with AD onset after the age of 40 or with atypical features. Dupilumab should be discontinued once CTCL confirmed, and methotrexate or phototherapy considered as alternatives. Cyclosporine and JAK inhibitors are considered unsuitable, and switching to other Th2‐targeting biologics is discouraged due to insufficient data.

**Conclusions:**

Dupilumab may unmask or exacerbate CTCL, particularly MF and SS. The consensus‐based recommendations offer practical guidance for the safe management of patients.

## Graphical abstract

Systematic review (51 studies; 547 patients) indicates dupilumab may unmask/worsen CTCL (mainly MF/SS). A French Delphi consensus recommends rigorous diagnostic workup, avoiding dupilumab in confirmed CTCL, discontinuing upon diagnosis, and favouring methotrexate or phototherapy. Cyclosporine, JAK inhibitors and switching to other Th2 biologics are discouraged.

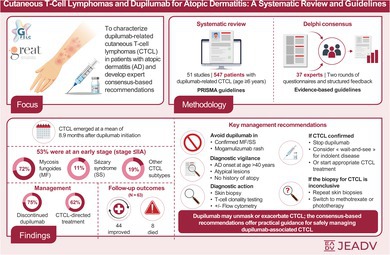

**Why was the study undertaken?**

This study was undertaken to clarify the potential association between dupilumab use and the emergence or worsening of cutaneous T‐cell lymphomas (CTCL), particularly mycosis fungoides and Sézary syndrome. The main research questions were whether dupilumab could unmask pre‐existing CTCL or accelerate its course, and how clinicians should manage diagnostic and therapeutic decisions in such scenarios.

**What does this study add?**

This study provides the largest systematic review to date, synthesizing data from 51 studies encompassing 547 patients with CTCL occurring during dupilumab therapy. It highlights the clinical characteristics, timelines and outcomes of these cases, and explores potential biological mechanisms linking IL‐4/IL‐13 blockade to malignant T‐cell proliferation. Importantly, it presents consensus‐based recommendations from a multidisciplinary expert panel, offering novel, evidence‐based guidance on the diagnostic workup, therapeutic decision‐making and risk stratification for patients considered for dupilumab.

**What are the implications of this study for disease understanding and/or clinical care?**

The study provides new insights into the pathogenesis of CTCL, suggesting that IL‐4Rα inhibition may unmask or worsen pre‐existing disease. Clinically, it emphasizes the importance of vigilance: patients with late‐onset or atypical atopic dermatitis should undergo thorough diagnostic evaluation, including histology and clonality testing, before and during dupilumab therapy. Consensus guidelines advise against using dupilumab in confirmed CTCL, recommend its discontinuation upon CTCL diagnosis and identify safer treatment alternatives that should help improve diagnostic rigour and patient safety.

## INTRODUCTION

Dupilumab is an IL‐4Rα‐targeting monoclonal antibody used widely to treat moderate‐to‐severe atopic dermatitis (AD). It works by blocking IL‐4 and IL‐13 signalling, thereby reducing inflammation and pruritus. Since its approval, however, rare but accumulating reports have raised concerns about the emergence of cutaneous T‐cell lymphomas (CTCL), particularly mycosis fungoides (MF) and Sézary syndrome (SS), in some patients receiving dupilumab for presumed AD.^1^, ^2^, ^3^, ^4^, ^5^ Furthermore, retrospective cohort studies have suggested an increased incidence of CTCL in dupilumab‐treated patients with AD compared to matched controls not receiving biologics.^6^, ^7^

Several potentially multifactorial mechanisms have been suggested that may explain the occurrence of CTCL in patients with AD treated with dupilumab. Although the exact biological mechanisms linking dupilumab with CTCL onset remain under investigation, one hypothetic one involves blockage of IL‐4 and IL‐13 signalling leading to an altered cutaneous immune microenvironment that favours clonal T‐cell expansion. In particular, increased bioavailability of IL‐13 may promote tumour cell proliferation through its facilitated engagement with IL‐13Rα2, a receptor expressed on malignant T cells that is not blocked by dupilumab.^8^, ^9^

Another hypothesis is that dupilumab unmasks rather than induces CTCL, particularly in cases where initial inflammatory presentations resemble AD.^10^, ^11^

Despite the emerging concerns over dupilumab use in such populations, establishing a causal relationship is challenging due to the rarity of CTCL, limitations in case documentation and the absence of systematic pre‐treatment histopathological and molecular assessment. To address these gaps, the French Cutaneous Lymphomas Group (GFELC) and the French Study Group on Atopic Eczema (GREAT) conducted a systematic review of published cases. This review aimed to characterize the clinical features, temporal dynamics and outcomes of CTCL in the context of dupilumab use, and to explore underlying pathogenic mechanisms. A modified delphi process then conducted on the obtained data helped develop consensus‐based recommendations to limit the use of dupilumab in ‘CTCL high‐risk’ patients and guide clinicians and pathologists in the diagnosis and management of CTCL arising during dupilumab therapy.

## MATERIALS AND METHODS

### First step: Systematic review

#### Literature search

A comprehensive and systematic literature search was conducted across PubMed, Web of Science and the Cochrane Library from inception to 2 January 2025, without language or geographic restrictions. The search strategy used the following Boolean terms: (‘lymphoma’ OR ‘lymphoproliferative disorders’ OR ‘cutaneous T‐cell lymphoma’ OR ‘Sezary’ OR ‘mycosis fungoides’) AND (‘dupilumab’).

#### Study population

All studies reporting on patients aged ≥6 years who were treated with dupilumab were considered for review.

#### Eligibility criteria

*Inclusion criteria*: Original articles reporting on patients aged ≥6 years and that met one or more of the following study designs: Phase I–III randomized controlled trials (RCTs), non‐randomized interventional studies, case–control studies, prospective or retrospective cohort studies, cross‐sectional or longitudinal studies, before‐and‐after studies, case series and case reports.

*Exclusion criteria* :Narrative or non‐systematic reviews, systematic reviews and meta‐analyses, conference abstracts or proceedings, editorials, commentaries, interviews, lectures, guidelines, consensus statements, newspaper articles, studies lacking full‐text availability and studies not involving human subjects.

#### Intervention

The intervention of interest was dupilumab.

#### Outcomes

The primary outcome was the occurrence of CTCL or any other primary cutaneous lymphoproliferative disorder following dupilumab exposure.

#### Study selection

Two investigators (MN and CD) independently screened all titles and abstracts. Discrepancies were resolved through discussion to reach consensus. Full‐text articles of potentially eligible studies were then independently reviewed by the same investigators. No disagreements occurred during study selection.

#### Data extraction

Data were independently collected by two investigators (MN and CD) using a standardized electronic data collection form. This systematic review adhered to the Cochrane Handbook for Systematic Reviews of Interventions^12^ and followed the PRISMA (Preferred Reporting Items for Systematic Reviews and Meta‐Analyses) guidelines.^13^

#### Statistical analysis

A descriptive analysis was performed for reported cases of CTCL and cutaneous lymphoproliferative disorders, as well as for the dermatological indications for dupilumab treatment. Categorical variables were reported as frequencies (percentages), while continuous variables were expressed as mean ± standard deviation (SD) or median with interquartile range (IQR).

#### Assessment of study quality

MN independently assessed study quality using different methods comprising: the recommendations of Murad et al. for case reports and case series^14^ focusing on four domains (selection, ascertainment, causality and reporting); the Appraisal tool for cross‐sectional studies (AXIS) recommendations for cross‐sectional studies from the BMJ Open^15^; the Newcastle–Ottawa Scale (NOS) for retrospective cohort studies^16^; STrengthening the Reporting of OBservational studies in Epidemiology (STROBE)^17^ and the Risk Of Bias In Non‐randomized Studies–of Interventions (ROBINS‐I)^18^ recommendations for pharmacovigilance studies.

#### Protocol registration

The study protocol was registered with PROSPERO international prospective register of systematic reviews (ID: CRD42025630079).

### Second step: Expert consensus methodology

Guidelines were developed by the French Study Group on Cutaneous Lymphomas (GFELC) and the Study Group on Atopic Dermatitis (GREAT), in accordance with the EQUATOR Network's recommendations for guideline development and the AGREE (Appraisal of Guidelines for Research and Evaluation) framework.^19^ The steering committee (FA, MN, ADM, MB, MT, SIHO, CD, DSS and MBB) appointed a multidisciplinary panel of 37 experts (18 from GFELC and 19 from GREAT), based on their expertise in CTCL and/or AD, to form the guidelines development group. The panel included 34 hospital‐based dermatologists, one dermatologist with both private and hospital‐based practice experience, and two pathologists, representing 26 institutions across two countries (France and Belgium). All the experts provided informed consent to participate and held voting rights.

The development process followed an expert consensus approach based on a modified delphi method.^20^ The steering committee defined the topics, questions and statements based on data from the literature review and their clinical experience. Two online meetings were held to finalize the list of statements for the first round of voting. Each questionnaire item was formulated as a declarative statement and rated using a 9‐point ordinal Likert‐type scale, where 7–9 indicated agreement. Participants were also invited to provide individual comments and submit new proposals for consideration. Before the first round of voting, the results of the systematic literature review were shared with all panel members to ensure that all experts had access to the same level of information before casting their votes. At each stage of the consensus process, individual responses were collected electronically from each panellist, and anonymized summary results were subsequently shared with the group. ‘Consensus’ was defined as greater than 75% agreement (i.e. a rating of ≥7) among panel members, and a median score of ≥7 with low response variability (standard deviation ≤2). Responses during the first round were shared among the expert panel, and the Steering Committee revised and reworded certain items to improve clarity before the second voting round. Items for which consensus had already been reached during the first round and that did not elicit questions or concerns from the panel were excluded from subsequent voting. The full delphi process thus consisted of two rounds of questionnaires and structured feedback.

## RESULTS

### Systematic review

**FIGURE 1:**
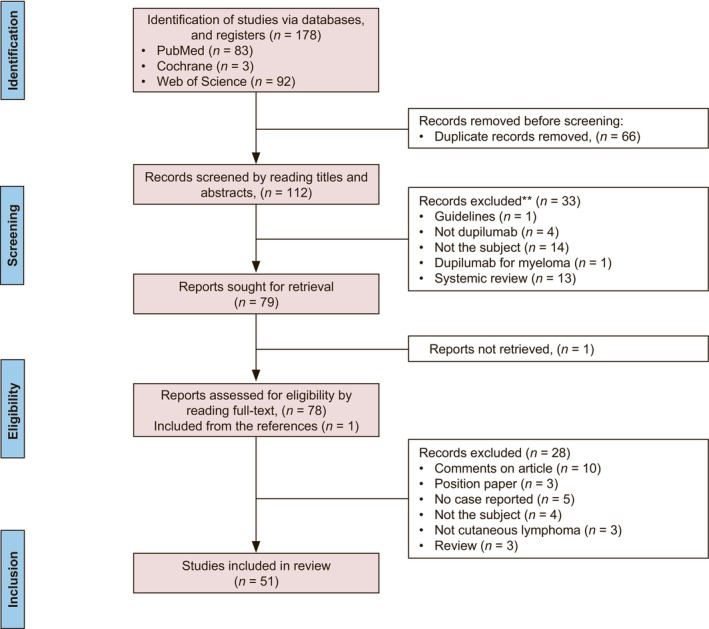
PRISMA 2020 flow diagram of the study selection process.

Of the 178 records provided by the search (Figure 1), duplicate removal yielded 112 for screening. We then excluded 33^21^, ^22^, ^23^, ^24^, ^25^, ^26^, ^27^, ^28^, ^29^, ^30^, ^31^, ^32^, ^33^, ^34^, ^35^, ^36^, ^37^, ^38^, ^39^, ^40^, ^41^, ^42^, ^43^, ^44^, ^45^, ^46^, ^47^, ^48^, ^49^, ^50^, ^51^, ^52^, ^53^ of these based on title and abstract content, a further 28^3^, ^4^, ^9^, ^10^, ^54^, ^55^, ^56^, ^57^, ^58^, ^59^, ^60^, ^61^, ^62^, ^63^, ^64^, ^65^, ^66^, ^67^, ^68^, ^69^, ^70^, ^71^, ^72^, ^73^, ^74^, ^75^, ^76^, ^77^ after a full‐text review and one final article due to the unavailability of its full text.^78^ One relevant article found in the references was added to the review.

In total, we included 51 studies: 2 cross‐sectional studies,^79^, ^80^ 2 retrospective chart reviews,^81^, ^82^ 6 retrospective cohort studies,^5^, ^6^, ^7^, ^83^, ^84^, ^85^ 2 pharmacovigilance studies,^86^, ^87^ 6 case series^1^, ^2^, ^88^, ^89^, ^90^, ^91^ and 33 case reports (Table 1).^11^, ^92^, ^93^, ^94^, ^95^, ^96^, ^97^, ^98^, ^99^, ^100^, ^101^, ^102^, ^103^, ^104^, ^105^, ^106^, ^107^, ^108^, ^109^, ^110^, ^111^, ^112^, ^113^, ^114^, ^115^, ^116^, ^117^, ^118^, ^119^, ^120^, ^121^, ^122^, ^123^ None of the included studies were randomized controlled trials. Most of the included studies were monocentric, while seven were multicentre. Twenty‐six studies were conducted in the United States, 12 in Europe, 12 in Asia and 1 in Brazil.

**TABLE 1:** Number of participants included by article type.

Type of article	Number of articles	Total of participants
Case report	33	32
Case series	6	46
Cross‐sectional study	2	12
Retrospective cohort study	6	166
Retrospective chart review	2	4
Pharmacovigilance study	2	287
Total	51	547

The total number of patients diagnosed with CTCL following exposure to dupilumab was 531. Additionally, we documented 12 cases of lymphoid reactions,^89^ 3 of parapsoriasis,^82^ 5 of angioimmunoblastic T‐cell lymphoma^87^, ^108^ and 1 of paraneoplastic lymphomatoid papuloerythroderma of Ofuji.^118^

The characteristics of the 547 participants are summarized in Table 2. Missing data were substantial, particularly in the two pharmacovigilance studies (*n* = 287; 53%), where individual‐level information was unavailable. Hereafter, we summarize the results based on the available data for each variable. The number of patients for whom the value is missing is provided in Table 2. The mean of patient age when available was 58 years (SD 11.5; range 10–85), and 47% were women. Among the patients treated with dupilumab for AD (*n* = 327) and for whom AD onset was recorded (*n* = 79), 59% had AD onset before the age of 40. Of the patients for whom treatment prior to dupilumab was known (*n* = 98), 70% had received systemic immunosuppressive therapy, most commonly cyclosporine (61%) and methotrexate (36%). Clinical change under dupilumab treatment reported for 126 patients was new skin lesions in 25% and a worsening of pre‐existing lesions in 75%, with a mean time to progression of 8.9 months (SD 5.1; range 0.5–27). Among the diagnoses reported (*n* = 146), MF was the most frequent (*n* = 103, 72%), followed by SS (*n* = 16, 11%) and other CTCL subtypes (*n* = 27, 19%), including eight cases of primary cutaneous CD30+ anaplastic large‐cell lymphoma and one case of TCRδ‐expressing epidermotropic lymphoma. Histological features at CTCL diagnosis were available for 74 patients (51% of *n* = 146 patients with CTCL diagnosis). Of these, 69 underwent skin TCR‐gene rearrangement analysis and clonal populations were identified in 51 cases (74% of *n* = 69). Blood T‐cell clonality testing reported in 17 patients (11.6% of n = 146) gave 10 positive results, while blood immunophenotyping was available in 43 patients (29.5% of n = 146). Among cases with staging data (*n* = 135), 71 patients (53%) were diagnosed at an early stage (≤ IIA).^124^

**TABLE 2:** Characteristics of CTCL and other cutaneous lymphoproliferative disorders reported during dupilumab therapy.

No. of participants	547
Age, N/A 256	
Mean +/SD	58.2 ± 11.5
Min–Max	10–85
Sex, Nb (%), N/A 328	
Female	103 (47)
Diagnosis before initiation of dupilumab, Nb (%), N/A 202	
AD	327 (95)
CTCL	6 (1.7)
Concomitant AD and CTCL	10 (2.9)
Others	2 (0.58)
Age at AD onset, Nb (%), N/A 258	
<40 years old	47 (59)
≥40 years old	32 (41)
Personal history of atopy, Nb (%), N/A 466	
Yes	45 (56)
Clinical phenotypes of AD, Nb (%), N/A 454	
Known	85 (16)
Histology performed before initiation of dupilumab, Nb (%), N/A 482	
Yes	50 (77)
No	15 (23)
Systemic treatment for AD before initiation of dupilumab Nb (%), N/A 449	
Yes	69 (70)
No	29 (30)
Methotrexate	25 (36)
Cyclosporine	42 (61)
Azathioprine	7 (10)
JAKi	2 (2.9)
Phototherapy	20 (29)
Biologics used for psoriasis^a^	9 (13)
Others	15 (22)
Controlled AD, Nb (%), N/A 430	
Yes	4 (3.7)
No	71 (65)
Initial improvement only	31 (29)
Time since dupilumab exposure and new skin lesions, in months, N/A 438	
Mean ± SD	8.9 ± 5.1
Time since dupilumab exposure and CTCL diagnosis, in months, N/A 416	
Mean ± SD	11.5 ± 5.4
Type of CTCL, Nb (%), N/A 404	
Mycosis fungoides	103 (72)
Sézary syndrome	16 (11)
Others	27 (19)
Disease stage, Nb (%), N/A 412	
Early stage^b^	71 (53)
Advanced stage^b^	64 (47)
Clinical change under dupilumab, Nb (%), N/A 421	
Worsening of previous lesions	94 (75)
New skin lesions	32 (25)
Clinical phenotypes of CTCL, Nb (%), N/A 438	
Available	109 (20)
Erythroderma	25 (23)
Palmoplantar keratoderma	8 (7.3)
Typical patches of mycosis fungoides	13 (12)
Follicular involvement	2 (1.8)
Peripheral lymphadenopathy	18 (17)
Others	69 (63)
Histology for CTCL diagnosis, Nb (%), N/A 473	
Characteristics available	74 (51)
Transformed CTCL	3 (4)
Retrospective diagnosis of CTCL on previous biopsy, Nb (%)	
Yes	5 (0.9)
No	28 (5.1)
*Not investigated*	*514 (94)*
Blood lymphocyte immunophenotyping performed, Nb (%), N/A 504	
Yes	43 (7.9)
Blood clonal TCR‐gene rearrangement, Nb (%), N/A 530	
Positive	10 (59)
Negative	7 (41)
Skin clonal TCR‐gene rearrangement, Nb (%), N/A 478	
Positive	51 (74)
Negative	18 (26)
Dupilumab discontinuation, Nb (%), N/A 468	
Yes	59 (75)
No	20 (25)
Systemic treatment for CTCL, Nb (%), N/A 439	
Yes	67 (62)
Methotrexate	16 (24)
Bexarotene and other retinoids	27 (40)
Mogamulizumab	4 (6.0)
Brentuximab	6 (9.0)
Chemotherapy	7 (10)
Extracorporeal photopheresis	7 (10)
Phototherapy	27 (40)
Radiotherapy	7 (10)
Haematopoietic stem cell transplantation	2 (3.0)
Other systemic treatments	8 (12)
Time at last follow‐up, in months, N/A 500	
Mean ± SD	12.9 ± 8.2
Evolution at last follow‐up, Nb (%), N/A 484	
Resolution	44 (70)
Persistence	11 (17)
Death	8 (13)

*Note*: Percentages are given among available data. Abbreviations: AD, atopic dermatitis; CTCL, cutaneous T‐cell lymphoma; JAKi, JAK inhibitors; Max, maximum; Min, minimum; N/A, not available; Nb, number; SD, standard deviation.

^a^ Guselkumab and secukinumab.

^b^ Early stage: ≤ stage IIA; advanced stage: ≥ stage IIB.

Among the 50 patients for whom skin biopsies had been performed before dupilumab initiation, 33 samples were re‐evaluated, leading to a retrospective diagnosis of CTCL in five cases.

Decision to discontinue dupilumab was made in 75% (*n* = 59) of the 79 cases with available data. Among patients with available information on CTCL management (*n* = 108), 62% (*n* = 67) received CTCL‐directed treatment. Reported therapies included bexarotene (*n* = 27, 40%), phototherapy (n = 27, 40%), methotrexate (*n* = 16, 24%), chemotherapy (*n* = 7, 10%), extracorporeal photopheresis (n = 7, 10%), radiotherapy (n = 7, 10%), brentuximab (n = 6, 9.0%), mogamulizumab (*n* = 4, 6.0%) and haematopoietic stem cell transplantation (n = 2, 3.0%). Eight patients received other treatments, such as romidepsine, interferon and pralatrexate.

Among the 63 patients for whom follow‐up data were available, 44 experienced partial or complete remission of skin lesions, 11 had persistent or stable diseaseand 8 died.

We performed additional analyses excluding studies without individual data, the results of which are presented in Tables S1 and S2.^2^, ^5^, ^6^, ^7^, ^82^, ^83^, ^84^, ^86^, ^87^, ^89^

The overall demographic and clinical characteristics were broadly consistent between the two datasets. However, Table S1, which includes only individual patient‐level data, features proportionally more advanced cases (62% vs 47%) and fatal outcomes (25% vs. 13%) compared with Table 2, possibly reflecting a publication bias whereby more severe or atypical cases are more likely to receive detailed reporting.

Concerning the quality of data measured using the criteria outlined in the materials and methods section above, 29 of the 39 case reports or series were poor and 10 were good. The overall quality of cross‐sectional studies was moderate, mainly due to small sample sizes and the monocentric nature of the studies. That of retrospective cohort studies was average due to questionable comparability between cohorts and insufficient detail provided on participant selection criteria. Retrospective chart reviews^125^ were generally of good quality, but data were missing regarding bias management and the training of evaluators.

For pharmacovigilance studies, we applied the STROBE^17^ and ROBINS‐I^18^ recommendations. Such studies are subject to inherent reporting bias (over‐ or under‐reporting), which can affect the reliability of the results. Additionally, the lack of access to individual data may have led to residual confounding bias.

### Expert consensus

**FIGURE 2:**
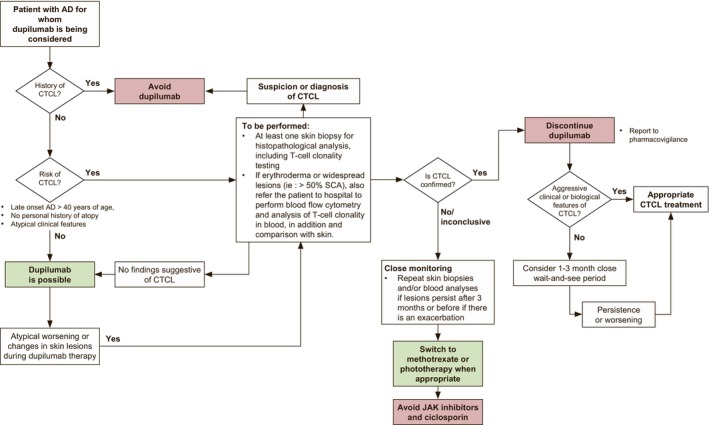
Algorithm for the assessment and management of dupilumab in patients with atopic dermatitis and suspected or confirmed cutaneous T‐cell lymphoma. AD, atopic dermatitis; CTCL, cutaneous T‐cell lymphoma; BSA, body surface area.

The final expert consensus voting on the use of dupilumab in suspected or confirmed CTCL showed a high level of agreement across most clinical scenarios (Table 3, Table S3). The panel addressed seven key topics, each containing focused questions on diagnosis, treatment decisions and follow‐up strategies for patients receiving dupilumab. A detailed account of the discussions and decisions is provided in Appendix S1. Based on these consensus statements, we developed a decision‐making algorithm for managing patients with AD in whom dupilumab is considered (Figure 2).

**TABLE 3:** Final expert consensus recommendations developed using a modified Delphi methodology.

Recommendation	Median score/9 (SD)	Consensus (% agreement)
1. Dupilumab use and risk of Cutaneous T‐cell Lymphoma (CTCL)		
1–1 Do you consider that dupilumab is more likely to unmask pre‐existing CTCL rather than cause it?	8.0 (1.62)	Yes (82.9%)
1–2 Do you consider that dupilumab may worsen pre‐existing CTCL?	8.0 (1.35)	Yes (80.0%)
2. Dupilumab Use in MF/SS Patients		
2–1 Should dupilumab be avoided in patients with mycosis fungoides (MF) or Sézary syndrome (SS)?	9.0 (0.80)	Yes (100.0%)
2–2 Should dupilumab be avoided in patients presenting mogamulizumab‐induced rashes?	8.0 (1.21)	Yes (91.3%)
3. Diagnostic Precautions Before Dupilumab in Adult‐Onset AD		
3–1 In patients with newly diagnosed atopic dermatitis (AD), should the possibility of CTCL be evoked before initiating dupilumab in the following clinical contexts?		
Not necessarily for all patients with AD	9.0 (1.31)	Yes (97.1%)
In adult patients over 40 years of age	8.0 (1.06)	Yes (94.1%)
In adult patients with no personal history of atopy	9.0 (1.00)	Yes (97.1%)
In patients presenting with atypical clinical features (e.g. distribution of lesions other than classic flexural localization, follicular lesions and alopecia, erythroderma, severe palmoplantar keratoderma, enlarged peripheral nodes)	9.0 (0.36)	Yes (100.0%)
3–2 When it is necessary to distinguish atopic dermatitis from CTCL, which of the following investigations should be conducted?		
At least one skin biopsy for histopathological analysis, including T‐cell clonality testing	9.0 (0.36)	Yes (100.0%)
If erythroderma or widespread lesions (i.e. > 50% SCA) are present, refer the patient to hospital to perform blood flow cytometry and analysis of T‐cell clonality in skin and blood.	9.0 (1.51)	Yes (91.4%)
4. Diagnostic Reassessment During Dupilumab Treatment		
4–1 In cases of atypical worsening or changes in skin lesions during dupilumab therapy, should the initial diagnosis of AD be reassessed?	9.0 (0.43)	Yes (100.0%)
4–2 In cases of atypical worsening or changes in skin lesions during dupilumab therapy, which of the following paraclinical investigations should be performed?		
At least one skin biopsy for histopathological analysis, including T‐cell clonality testing	9.0 (0.36)	Yes (100.0%)
If erythroderma or widespread lesions (i.e. > 50% body surface area BSA) are present, refer the patient to hospital to perform blood flow cytometry and analysis of T‐cell clonality in skin and blood.	9.0 (1.44)	Yes (94.3%)
5. Management When CTCL Is Diagnosed		
If CTCL is confirmed by skin biopsy during dupilumab treatment, what is the appropriate management approach?		
Discontinue dupilumab immediately in all cases.	9.0 (0.88)	Yes (97.1%)
After dupilumab discontinuation, if clinical features are non‐aggressive, consider a 1–3‐month ‘wait‐and‐see’ with only symptomatic approach and close monitoring	9.0 (1.43)	Yes (91.2%)
If there is no improvement or clinical worsening after dupilumab discontinuation and an initial ‘wait‐and‐see’ period, appropriate CTCL treatment should be initiated	9.0 (1.56)	Yes (88.2%)
After dupilumab discontinuation, in case of clinical features suggesting an aggressive behaviour, appropriate CTCL treatment should be initiated	9.0 (0.29)	Yes (100.0%)
Report to pharmacovigilance authorities.	9.0 (0.29)	Yes (100.0%)
6. Inconclusive Biopsy for CTCL		
In cases of atypical worsening or changes in skin lesions during dupilumab therapy, and the skin biopsy is inconclusive for CTCL, what is the appropriate management approach?		
Close monitoring in a clinical setting is required	9.0 (0.47)	Yes (100.0%)
Repeat skin biopsies and/or blood analyses if lesions persist after 3 months or before if there is an exacerbation	9.0 (0.32)	Yes (100.0%)
Consider switching dupilumab to methotrexate when appropriate	8.5 (1.59)	Yes (91.2%)
Consider switching dupilumab to phototherapy when appropriate	8.0 (1.00)	Yes (97.1%)
Consider switching dupilumab to tralokinumab if no other therapeutic options are suitable based on the clinical context	7.0 (2.13)	No (51.6%)
Consider switching dupilumab to lebrikizumab if no other therapeutic options are suitable based on the clinical context	7.0 (2.23)	No (61.3%)
Avoid switching dupilumab to JAK inhibitors	8.0 (1.28)	Yes (84.8%)
Avoid switching dupilumab to cyclosporine	9.0 (1.48)	Yes (91.2%)
7. Data Collection and Research		
Is the creation of a dedicated database including histological findings and patient outcomes appropriate to better understand this clinical context?	9.0 (0.47)	Yes (100.0%)

## DISCUSSION

Dupilumab is one of the most widely used systemic treatments for moderate‐to‐severe AD. Its mechanism of action is potentially relevant for MF through its action on the Th2 pathway and on pruritus, which is an issue in MF/SS. It has also occasionally been proposed for mogamulizumab‐associated rash (MAR)^99^ or for the management of severe pruritus in palliative care settings.^96^ However, reports of the emergence of CTCL under dupilumab are an important concern. While data on these cases are heterogeneous, with missing information for large numbers of patients, as shown by our systematic review, some characteristics may be identified.

Clinical patterns have been observed with CTCL emergence, one of which is late‐onset AD as described recently by Cabrera‐Perez et al. analysing Food and Drug Administration's Adverse Event Reporting System between 2017 and 2023.^87^ Others include absence of personal atopic history, resistance to dupilumab and development of new or atypical lesions during treatment. The outcome of CTCL is not always specified but some fatal outcomes with inefficacy of treatments have been reported (13% of the patients with documented outcome). However, in a matched controlled cohort study including 27 patients, five patients who developed MF/SS during dupilumab treatment had a natural history similar to that of age‐ and stage‐matched controls.^5^ Also, in our systematic review, more than half of the reported cases were diagnosed at early stages. Furthermore, MF‐like infiltrates that mimic CTCL have also been described in dupilumab‐treated patients who showed complete or partial regression of their lesions after treatment withdrawal.^89^ Such spontaneous improvement following discontinuation of dupilumab without the need for CTCL‐specific therapy has been reported previously.^3^, ^89^ Although outcome data remain heterogeneous and often incomplete, these observations support the hypothesis of a reversible, drug‐related process in some patients and justify, in indolent or early‐stage disease, an initial period of clinical monitoring (‘wait‐and‐see’ approach) before initiating cytotoxic or systemic treatments.

Our search did not retrieve data from randomized controlled trials, which often enrol a highly selected group of patients that may differ from those encountered in real‐life settings. Recently, only a single case of cutaneous lymphoma was reported across pooled phase III and open‐label extension studies, representing over 7000 patient‐years of dupilumab exposure.^126^

Some patients developing CTCL under dupilumab may have had undiagnosed lymphoma at baseline. While early MF can mimic AD, CTCL may occur concomitantly with AD, thereby complicating diagnosis. In our review, retrospective reassessment of pre‐dupilumab biopsies confirmed CTCL in several cases, highlighting the potential for baseline misclassification. Thus, in some patients, dupilumab may have unmasked or accelerated pre‐existing CTCL rather than inducing de novo disease. Pathophysiological mechanisms that may explain this involve the blockade of IL‐4R and IL‐13Rα1 heterodimer, which leads to an increase in the availability of IL‐13 for binding to IL‐13Rα2‐expressing lymphoma tumour cells, potentially driving their proliferation.^8^ An additional mechanism recently suspected is that the keratinocytes would normally absorb excess IL13 and that this is blocked by dupilumab, leaving excess IL13 to bind to and activate the neoplastic T‐cells.^87^

Considering these findings, caution is warranted regarding other biologics that act through IL‐4Rα blockade. The potential risk associated with other Th2‐targeting biologics remains uncertain to date. Two more recently approved drugs, tralokinumab and lebrikizumab, do have slightly different mechanisms of action; however, current evidence fails to substantiate their use as safer alternatives to dupilumab in cases of suspected or potential CTCL. Indeed, one recent case report described the unmasking of MF following tralokinumab treatment,^127^ raising concerns about the potential for similar outcomes. The long‐term safety profiles of these more recently approved drugs, particularly in patients at risk for CTCL, remain poorly characterized and the potential emergence of additional cases should not be excluded. The results of the delphi exercise on this topic (part of Topic 6) reflect this uncertainty and the necessary caution exercised by the expert panel.

Similarly, broad expert consensus cautioned against switching from dupilumab to Janus kinase (JAK) inhibitors upon inconclusive skin biopsy with regard to CTCL. The JAK/signal transducer and activator of transcription (STAT) pathway plays a critical role in the pathogenesis of CTCL, with recurrent mutations and copy number variations identified in JAK1, JAK3, STAT3 and STAT5B across several CTCL subtypes, including MF and SS.^128^, ^129^ These molecular aberrations result in constitutive activation of the JAK/STAT cascade, promoting neoplastic T‐cell proliferation and survival.^130^, ^131^ Recent clinical and translational data support the potential utility of JAK inhibitors such as ruxolitinib and cerdulatinib in MF and SS.^132^, ^133^ A recent case report described two elderly patients with refractory MAR who responded successfully to treatment with the selective JAK1 inhibitor upadacitinib.^134^ However, longer follow‐up and more robust quantitative data are needed to assess the risk of CTCL recurrence in these patients and to establish the long‐term safety of JAK inhibition in this context. Several cases of de novo CTCL or relapse have been reported in patients receiving JAK inhibitors for autoimmune diseases or chronic graft‐versus‐host disease, suggesting a possible lymphomagenic role in immunocompromised individuals.^135^, ^136^, ^137^ These findings underscore the dual nature of JAK inhibition in CTCL: a promising targeted therapy in selected cases, but requiring careful patient selection, molecular profiling and long‐term monitoring. Altogether, while current evidence supports the therapeutic rationale for JAK inhibition in CTCL, further registries and prospective, biomarker‐stratified studies are essential to refine indications and clarify the long‐term safety of these agents in CTCL.

Interestingly, cases of CTCL have also been described in patients receiving biologic therapies for non‐AD indications, particularly psoriasis. In this context, misdiagnosis should also be considered, as biologic therapy may unmask an underlying or previously unrecognized CTCL, similar to observations in AD. In a French multicentre registry, most cases occurred under anti‐TNF‐α agents, and psoriasis was the main underlying condition.^138^ More recently, a systematic review of psoriasis biologic trials and registries confirmed that CTCL remains rare overall, with most reports involving TNF‐α inhibitors and none observed with IL‐23 inhibitors.^139^ These findings suggest that CTCL emergence during biologic therapy is not limited to type‐2‐targeting agents and may involve other immune pathways such as Th17/IL‐23, reinforcing the need for careful clinical and histopathologic evaluation before and during biologic treatment.

The hypothesis that dupilumab may unmask rather than induce CTCL is supported by the difficulties encountered diagnosing certain MFs and the MF/AD similarities or even common pathogenesis. Moreover, an ‘increased risk’ of lymphoma, particularly cutaneous lymphoma, has been identified in AD,^140^, ^141^, ^142^, ^143^ but dupilumab seems to have an additional impact. Compared to patients with AD either left untreated or who had never taken dupilumab, those treated with dupilumab were shown to have a higher risk of developing CTCL in a recent TrinetX database analysis and a large real‐world pharmacovigilance study of the FDA adverse event reporting system.^6^, ^86^

Cutaneous lymphoma in patients treated with dupilumab has mainly been described in patients with AD or related inflammatory dermatoses, as shown in our review (> 95% of cases) and in a recent study on FDA reports.^86^ The potential for dupilumab to unmask or exacerbate CTCL in patients without a history of AD remains unclear and warrants further investigation. A recent US population‐based cohort study assessed the risk of lymphoma in asthma patients treated with dupilumab.^144^ Among over 14,900 matched dupilumab users and controls, a significantly increased risk of lymphoma was observed in the dupilumab group (HR = 1.79), particularly for T and natural killer cell lymphomas. Notably, the risk for CTCL, including MF and SS, was markedly elevated (HRs >5). These findings persisted even after excluding patients with AD and adjusting for baseline characteristics. Although all‐cause mortality was lower among dupilumab users, the results raise safety concerns, especially regarding CTCL development. However, although hazard ratios for CTCL and peripheral T‐cell lymphoma (PTCL) were high, the absolute number of cases was low (≤10 in some subgroups), thus limiting the statistical power. Moreover, AD may have been underdiagnosed or misclassified, potentially introducing selection bias. Overall, these findings suggest that while pre‐existing cutaneous inflammation may not be a prerequisite for CTCL development in patients treated with dupilumab, it could significantly facilitate or accelerate the malignant process. In other words, dupilumab may instigate the underlying mechanism of action, while a pro‐inflammatory skin environment might enhance susceptibility to lymphomagenesis or unmask latent disease.

Considering the current widespread use of dupilumab for AD, providing clear practical guidelines for clinicians on its use to treat AD in different patient populations, particularly concerning CTCL, was of utmost importance.

In summary, this systematic review and expert consensus underscore the need for greater caution when using dupilumab in patients at risk for CTCL. Current evidence suggests that dupilumab may unmask latent disease or worsen existing CTCL and therefore should be initiated only in cases of typical AD. Clinicians should perform comprehensive diagnostic evaluations in adults presenting with atypical, treatment‐resistant or late‐onset dermatitis, both before initiating and during dupilumab therapy. Our guidelines development process demonstrated a robust level of expert consensus across key clinical decision points, emphasizing the need for diagnostic rigour cautious therapeutic choices and improved data collection.

## AUTHOR CONTRIBUTIONS

Florent Amatore coordinated the expert consensus process and contributed to manuscript writing. Madeleine Neildez conducted the systematic literature review and contributed to manuscript writing. Adèle de Masson contributed to manuscript writing. Maxime Battistella contributed to manuscript writing. Marie Tauber contributed to manuscript writing. Saskia Ingen‐Housz‐Oro contributed to manuscript writing. Catherine Droitcourt conducted the systematic literature review and contributed to manuscript writing. Delphine Staumont‐Sallé contributed to manuscript writing and provided senior oversight and critical guidance. Marie Beylot‐Barry coordinated the expert consensus process, contributed to manuscript writing and provided senior oversight and critical guidance.

## FUNDING INFORMATION

None.

## CONFLICT OF INTEREST STATEMENT

MBB: Received honorarium from Kyowa Kirin and Recordati and a research grant from Kyowa Kirin and Almirall. SIHO: No conflict of interest. CD: Has received speaker honorarium from Sanofi‐Genzyme, Eli Lilly and Co, AbbVie and UCB‐Pharma. FA: Received speaker honorarium from Kyowa Kirin, Sanofi‐Genzyme, Leo Pharma and Almirall. DSS: Investigator, consultant and speaker for AbbVie, Almirall, Eli‐Lilly, Leo Pharma, Pfizer and Sanofi‐Regeneron. MT: Investigator, consultant and speaker for AbbVie, Almirall, Leo Pharma, Pfizer, Sanofi‐Regeneron and Galderma. MB: received honorarium from Kyowa kirin, Takeda, Recordati, BMS, MSD, Innate Pharma, Eli Lilly, Regeneron and Sanofi. ADM: Received honoraria from Kyowa Kirin, Recordati, Therakos, Helsinn, Takeda, Almirall, Novartis and research grants from Kyowa Kirin and Almirall. MN: no conflict of interest.

## ETHICAL APPROVAL

Not applicable.

## ETHICS STATEMENT

Not applicable.

## Supporting information


Data S1.

## Data Availability

All study‐related data are available upon request to the corresponding author.
