# Supplementary material for: Cutaneous T‐cell lymphomas and dupilumab for atopic dermatitis: A systematic review and expert consensus
Source: J Eur Acad Dermatol Venereol. 2026 Mar 12;40(8):1344–58. doi: 10.1111/jdv.70407 (PMC13425264; doi:10.1111/jdv.70407)
Supplement: Supplementary file 1 — Data S1. [file JDV-40-1344-s001.docx]

**Appendix S1**

**Summary of expert consensus discussions and decisions on dupilumab use**

**in suspected or confirmed CTCL**

In Topic 1, the panel largely agreed that dupilumab is more likely to unmask pre-existing CTCL than cause it, and that it may worsen pre-existing CTCL. These findings suggest a strong perception among experts that dupilumab may interfere with disease expression in patients with occult CTCL.

Topic 2 addressed dupilumab use in patients with confirmed CTCL or mogamulizumab-induced rashes (MAR). There was unanimous consensus to avoid dupilumab in patients with MF/SS and strong agreement against its use in MAR. Because dupilumab modulates Th2 responses without fully restoring immune surveillance against malignant T cells, its use in MAR could exacerbate clonal proliferation. In this topic, the Steering Committee chose not to include a specific recommendation addressing the management of severe pruritus in palliative care settings. This decision reflects the recognition that such situations require highly individualized clinical judgment, often based on patient-specific goals, comorbidities, and quality-of-life considerations. While some panelists acknowledged that dupilumab might offer relief in selected palliative scenarios, especially when conventional treatments have failed, the committee concluded that the current evidence base is insufficient to support a standardized recommendation.

In Topic 3, focusing on diagnostic precautions before initiating dupilumab in adult-onset AD, there was again near-universal consensus that clinicians should consider excluding CTCL in any patient with suspected “AD” who is over 40 years of age, lacks prior atopy, or presents with atypical clinical features. The rationale for selecting a 40-year age cut-off was based on epidemiological and clinical considerations. While the average age at diagnosis of dupilumab-associated CTCL is approximately 58 years, symptoms, presenting as AD may begin several years before a formal diagnosis is established. The 40-year threshold was chosen as a pragmatic compromise between the typical age of adult-onset AD (around 23 years) and that of very-late-onset forms, which tend to appear closer to 60 years.^1-6^ Experts agreed to recommend at least one skin biopsy with histopathology and T-cell clonality testing, and referral for blood analysis and flow cytometry in cases of erythroderma or widespread lesions (> 50% of BSA). One panelist raised concerns about the feasibility of routine clonality testing in non-academic settings. However, the steering committee emphasized that T-cell clonality analysis can be performed on biopsies taken outside hospitals, as paraffin blocks and slides can be referred to expert centers. Moreover, assessing T-cell clonality is essential for the diagnosis of CTCL in such complex cases.

Topic 4 addressed how to manage unexpected clinical changes in a patient with AD treated with dupilumab. In the event of atypical worsening, (e.g., modified distribution of lesions with unusual localization, follicular lesions and alopecia, erythroderma, severe palmoplantar keratoderma, enlarged peripheral node), experts strongly recommended diagnostic reassessment, including repeated skin biopsies with clonality testing. In the presence of erythroderma or widespread lesions, referral to a hospital setting was advised to allow blood flow cytometry and clonality analysis of both skin and blood samples.

Topic 5 dealt with confirmed CTCL diagnosis under dupilumab. There was near-unanimous agreement that dupilumab should be discontinued immediately. Most supported a close wait-and-see approach (+/- topical treatment such as topical steroids) in non-aggressive cases and the initiation of CTCL-specific treatment if symptoms persisted or worsened. All respondents agreed that aggressive cases warrant immediate appropriate treatment.

In Topic 6, focusing on inconclusive biopsy results, there was universal consensus on the need for close clinical monitoring and repeated investigations as needed. Switching dupilumab to methotrexate or phototherapy was strongly supported, while switching to cyclosporine was strongly discouraged. The panel expressed strong caution regarding the use of tralokinumab and lebrikizumab in patients with suspected or possible CTCL, citing the lack of robust data to support their safety. Despite their slightly different mechanisms, experts considered that current evidence was insufficient to consider them as safer alternatives, and no consensus was reached on switching to either agent. Opinions on JAK inhibitors were more divided: while many recommended caution due to potential risks in latent CTCL, others noted their investigation in CTCL trials and potential in selected cases after multidisciplinary discussion. Overall, most experts advised against switching dupilumab to JAK inhibitors.

Finally, in Topic 7, all panelists agreed on the need to create a dedicated registry to collect clinical and histopathological data and better characterize outcomes in this setting.

1. Sigg N, Leducq S, Barbarot S, Salle DS, Ertus C, Hurson C, *et al.* French guidelines for the management of atopic dermatitis. *J Eur Acad Dermatol Venereol JEADV* 2025.

2. Bieber T, D’Erme AM, Akdis CA, Traidl-Hoffmann C, Lauener R, Schäppi G, *et al.* Clinical phenotypes and endophenotypes of atopic dermatitis: Where are we, and where should we go? *J Allergy Clin Immunol* 2017;**139**:S58–64.

3. Wollenberg A, Kinberger M, Arents B, Aszodi N, Barbarot S, Bieber T, *et al.* European Guideline (EuroGuiDerm) on atopic eczema: Living update. *J Eur Acad Dermatol Venereol JEADV* 2025.

4. Zhou L, Leonard A, Pavel AB, Malik K, Raja A, Glickman J, *et al.* Age-specific changes in the molecular phenotype of patients with moderate-to-severe atopic dermatitis. *J Allergy Clin Immunol* 2019;**144**:144–56.

5. Lee HH, Patel KR, Singam V, Rastogi S, Silverberg JI. A systematic review and meta-analysis of the prevalence and phenotype of adult-onset atopic dermatitis. *J Am Acad Dermatol* 2019;**80**:1526-1532.e7.

6. Silverberg JI, Vakharia PP, Chopra R, Sacotte R, Patel N, Immaneni S, *et al.* Phenotypical Differences of Childhood- and Adult-Onset Atopic Dermatitis. *J Allergy Clin Immunol Pract* 2018;**6**:1306–12.

| **Type of article** | **Number of articles** | **Total of participants** |
| --- | --- | --- |
| Case report | 32 | 32 |
| Case series | 4 | 14 |
| Cross-sectional study | 2 | 12 |
| Retrospective cohort study | 1 | 2 |
| Retrospective chart review | 1 | 1 |
| Pharmacovigilance study | 0 | 0 |
| Total | 40 | 61 |

**Suppl Table 1:** Number of participants included by article type (only data from studies with individual patient information)

| **Nb of participants** | **61** |
| --- | --- |
| **Age, N/A 0** | |
| Mean +/SD | 57.3 +/- 14.2 |
| Min-Max | 26-85 |
| **Sex,** **Nb (%), N/A 0** | |
| Female | 21 (34.4) |
| **Diagnosis before initiation of dupilumab, Nb (%), N/A 1** | |
| AD | 50 (83.3) |
| CTCL | 6 (10.0) |
| Concomitant AD and CTCL | 2 (3.3) |
| Others | 2 (3.3) |
| **Age at AD onset, Nb (%), N/A 15** | |
| < 40 years old | 15 (40.5) |
| >= 40 years old | 22 (59.5) |
| **Personal history of atopy, Nb (%), N/A 29** | |
| Yes | 21 (65.6) |
| **Clinical phenotypes of AD, Nb (%), N/A 22** | |
| Known | 30 (57.7) |
| **Histology performed before initiation of dupilumab, Nb (%), N/A 23** | |
| Yes | 32 (84.2) |
| No | 6 (15.8) |
| **Systemic treatment for AD before initiation of dupilumab Nb (%), N/A 14** | |
| Yes | 31 (66.0) |
| No | 16 (34.0) |
| Methotrexate | 11 (23.4) |
| Cyclosporine | 9 (19.1) |
| Azathioprine | 5 (10.6) |
| JAKi | 2 (4.3) |
| Phototherapy | 14 (29.8) |
| Biologics used for psoriasis* | 4 (8.5) |
| Others | 9 (19.1) |
| **Controlled AD, Nb (%), N/A 7** | |
| Yes | 4 (8.89) |
| No | 28 (62.2) |
| Initial improvement only | 13 (28.9) |
| **Time since dupilumab exposure and new skin lesions, in months, N/A 24** | |
| Mean +/- SD | 6.08 +/- 6.10 |
| **Time since dupilumab exposure and CTCL diagnosis, in months, N/A 31** | |
| Mean +/- SD | 10.2 +/- 7.72 |
| **Type of CTCL, Nb (%), N/A 6** | |
| Mycosis fungoides | 36 (65.5) |
| Sezary syndrome | 14 (25.5) |
| Others | 7 (12.7) |
| **Disease stage, Nb (%), N/A 24** | |
| Early-stage^§^ | 14 (37.8) |
| Advanced-stage^§^ | 23 (62.2) |
| **Clinical change under dupilumab, Nb (%), N/A 16** | |
| Worsening of previous lesions | 25 (55.6) |
| New skin lesions | 20 (44.4) |
| **Clinical phenotypes of CTCL, Nb (%), N/A 6** | |
| Available | 55 (90.2 |
| Erythroderma | 19 (34.5) |
| Palmoplantar keratoderma | 7 (12.7) |
| Typical patches of mycosis fungoides | 13 (23.6) |
| Follicular involvement | 1 (1.8) |
| Peripheral lymphadenopathy | 18 (32.7) |
| Others | 22 (40.0) |
| **Histology for CTCL diagnosis, Nb (%), N/A 23** | |
| Characteristics available | 38 (62.3) |
| Transformed CTCL | 3 (7.9) |
| **Retrospective diagnosis of CTCL on previous biopsy, Nb (%)** | |
| Yes | 2 (10.5) |
| No | 17 (89.5) |
| *Not investigated* | *42 (68.9)* |
| **Blood lymphocyte immunophenotyping performed, Nb (%), N/A 37** | |
| Yes | 24 (39.3) |
| **Blood clonal TCR-gene rearrangement, Nb (%), N/A 44** | |
| Positive | 10 (58.8) |
| Negative | 7 (41.2) |
| **Skin clonal TCR-gene rearrangement, Nb (%), N/A 36** | |
| Positive | 14 (56.0) |
| Negative | 11 (44.0) |
| **Dupilumab discontinuation, Nb (%), N/A 18** | |
| Yes | 38 (88.4) |
| No | 5 (11.6) |
| **Systemic treatment for CTCL, Nb (%), N/A 15** | |
| Yes | 35 (76.1) |
| Methotrexate | 5 (10.9) |
| Bexarotene and other retinoids | 12 (26.1) |
| Mogamulizumab | 4 (8.7) |
| Brentuximab | 6 (13.0) |
| Chemotherapy | 7 (15.2) |
| Extracorporeal photopheresis | 7 (15.2) |
| Phototherapy | 13 (28.3) |
| Radiotherapy | 7 (15.2) |
| Hematopoietic stem cell transplantation | 2 (4.3) |
| Other systemic treatments | 8 (17.4) |
| **Time at last follow-up, in months, N/A 44** | |
| Mean +/- SD | 11.06 +/- 8.7 |
| **Evolution at last follow-up, Nb (%), N/A 33** | |
| Resolution | 11 (39.3) |
| Persistence | 10 (35.7) |
| Death | 7 (25) |

**Suppl Table 2:** Characteristics of CTCL and other cutaneous lymphoproliferative disorders reported during dupilumab therapy (only data from studies with individual patient information)

Abbreviations: AD, atopic dermatitis; CTCL, cutaneous T-cell lymphoma; JAKi, JAK inhibitors; Max, maximum; Min, minimum; N/A, not available; Nb, number; SD, standard deviation.

*Guselkumab, secukinumab

§Early-stage: ≤ stage IIA ; advanced-stage: ≥ stage IIB

Percentages are given among available data.

| **Questions and/or Statements** | **Consensus**  **(≥ 75% agreement)**  **after the 1st round** | **Modified statement for the second round of consensus voting** |
| --- | --- | --- |
| 1-1 Do you consider that dupilumab is more likely to unmask pre-existing CTCL rather than cause it? | **Yes (82.9 %)** | *Second round not required* |
| 1-2 Do you consider that dupilumab may worsen pre-existing CTCL? | **Yes (80.0%)** | *Second round not required* |
| 2-1 Should dupilumab be avoided in patients with mycosis fungoides (MF) or Sézary syndrome (SS)? | **Yes (100.0%)** | *Second round not required* |
| 2-2 Should dupilumab be avoided in patients presenting mogamulizumab-induced rashes? | **Yes (91.3%)** | *Second round not required. The steering committee decided not to include any statement regarding the management of severe pruritus in palliative settings. This point will be addressed in the Discussion section of the article* |
| 3-1 In patients with newly diagnosed atopic dermatitis (AD), should the possibility of CTCL be excluded before initiating dupilumab in the following clinical contexts? | | *Second round not required. However, the question will be rephrased to improve clarity*  *“3-1*  *In patients with newly diagnosed atopic dermatitis (AD), should the possibility of CTCL be evoked before initiating dupilumab in the following clinical contexts?”* |
| Not necessary for all patients with AD | **Yes (97.1%)** | *Second round not required. « Necessary » will be replaced by « necessarily » to improve clarity.* |
| In adult patients over 40 years of age | **Yes (94.1%)** | *Second round not required. The rationale for selecting a 40-year age cut-off will be detailed in the Discussion section of the article.* |
| In adult patients with no personal history of atopy | **Yes (97.1%)** | *Second round not required* |
| In patients presenting with atypical clinical features (e.g., distribution of lesions other than classic flexural localization, follicular lesions and alopecia, erythroderma, severe palmoplantar keratoderma, enlarged peripheral nodes) | **Yes (100.0%)** | *Second round not required* |
| 3-2 When confirmation of atopic dermatitis (AD) is required, to exclude CTCL, which of the following paraclinical investigations should be performed? | | **Refined version of the question:**  **“3-2**  **When it is necessary to distinguish atopic dermatitis from CTCL, which of the following investigations should be conducted?** |
| At least one skin biopsy for histopathological analysis. | **Yes (100.0%)** | **At least one skin biopsy for histopathological analysis, including T-cell clonality testing** |
| At least one skin biopsy for T-cell clonality analysis. | **Yes (76.5%)** |  |
| If erythroderma or widespread lesions (ie : > 50% SCA) are present, refer the patient to hospital to perform blood flow cytometry and analysis of T-cell clonality in skin and blood. | **Yes (91.4%)** | *Second round not required* |
| 4-1 If AD worsens or lesions change atypically during dupilumab treatment, should the diagnosis of AD be reassessed? | **Yes (100.0%)** | *Second round not required. However, the question will be rephrased to improve clarity*  *4-1 In cases of atypical worsening or changes in skin lesions during dupilumab therapy, should the initial diagnosis of AD be reassessed?* |
| 4-2: If AD worsens or lesions change atypically during dupilumab treatment, which of the following paraclinical investigations should be performed? | | **4-2 In cases of atypical worsening or changes in skin lesions during dupilumab therapy, which of the following paraclinical investigations should be performed?** |
| At least one skin biopsy for histopathological analysis. | **Yes (100.0%)** | **At least one skin biopsy for histopathological analysis, including T-cell clonality testing** |
| At least one skin biopsy for T-cell clonality analysis. | **Yes (94.3%)** |  |
| If erythroderma or widespread lesions (ie : > 50% body surface area BSA) are present, refer the patient to hospital to perform blood flow cytometry and analysis of T-cell clonality in skin and blood. | **Yes (94.3%)** | *Second round not required* |
| 5-1 If CTCL is confirmed by skin biopsy during dupilumab treatment, what is the appropriate management approach? | |  |
| Discontinue dupilumab immediately in all cases. | **Yes (97.1%)** | *Second round not required* |
| After dupilumab discontinuation, if clinical features are non-aggressive, consider a 1–3 month “wait-and-see” with only symptomatic approach and close monitoring | **Yes (91.2%)** | *Second round not required* |
| If there is no improvement or clinical worsening after dupilumab discontinuation and an initial 'wait-and-see' period, appropriate CTCL treatment should be initiated | **Yes (88.2%)** | *Second round not required* |
| After dupilumab discontinuation, in case of clinical features suggesting an aggressive behavior, appropriate CTCL treatment should be initiated | **Yes (100.0%)** | *Second round not required* |
| Report to pharmacovigilance authorities. | **Yes (100.0%)** | *Second round not required* |
| 6-1 If AD worsens or lesions change atypically under dupilumab and the skin biopsy is inconclusive for CTCL, what is the appropriate management approach? | |  |
| Close monitoring in a clinical setting is required | **Yes (100.0%)** | *Second round not required* |
| Repeat skin biopsies and/or blood analyses if lesions persist after 3 months or before if there is an exacerbation | **Yes (100.0%)** | *Second round not required* |
| Consider switching dupilumab to tralokinumab when appropriate | **No (43.3%)** | **Consider switching dupilumab to tralokinumab if no other therapeutic options are suitable based on the clinical context** |
| Consider switching dupilumab to lebrikizumab when appropriate | **No (50.0%)** | **Consider switching dupilumab to lebrikizumab if no other therapeutic options are suitable based on the clinical context** |
| Consider switching dupilumab to Jak-inhibitors when appropriate | **No (39.4%)** | **Avoid switching dupilumab to JAK inhibitors** |
| Consider switching dupilumab to cyclosporine when appropriate | **No (18.2%)** | **Avoid switching dupilumab to cyclosporine** |
| Consider switching dupilumab to methotrexate when appropriate | **Yes (91.2%)** | *Second round not required* |
| *Added statement for the 2^nd^ round of voting* | | **Consider switching dupilumab to phototherapy when appropriate.** |
| 7-1 Is the creation of a dedicated database including histological findings and patient outcomes appropriate to better understand this clinical context? | **Yes (100.0%)** | *Second round not required* |

**Suppl. Table 3.** Results of the first round of expert consensus voting.
